# Impact of hypophosphatemia on outcome of patients in intensive care unit: a retrospective cohort study

**DOI:** 10.1186/s12871-019-0746-2

**Published:** 2019-05-24

**Authors:** Lichun Wang, Chaoxing Xiao, Lei Chen, Xiaofei Zhang, Qiuye Kou

**Affiliations:** Department of Intensive Care Unit, The Sixth Affiliated Hospital of Sun Yat-Sen University, Guangzhou, 510655 China

**Keywords:** Hypophosphatemia, 28-day mortality, Critical ill

## Abstract

**Background:**

Hypophosphatemia generally occurs in Intensive Care Units (ICUs), but its impact is often ignored. The aim of this study was to investigate whether hypophosphatemia can be a risk factor for ICU 28-day mortality.

**Methods:**

A single-center retrospective cohort study was conducted by collecting data from 1073 patients admitted to general ICU and then presented to the Sixth Affiliated Hospital, Sun Yat-sen University (Guangzhou City, Guangdong Province, China) from 1 January 2016 to 31 December 2017. The patients were divided into a normal control group (serum phosphate levels 0.80–1.60 mmol/L) and a hypophosphatemia group (serum phosphate levels < 0.80 mmol/L), based on the concentration of phosphorus at the time of ICU admission. The association between phosphate levels and ICU 28-day mortality was evaluated by binary logistic regression analysis. Multivariate logistic regression was employed to predict the ICU 28-day mortality.

**Results:**

The cohort included 946 patients with a median phosphate concentration of 0.77 mmol/L (interquartile range 0.55–1.03 mmol/L). Patients with hypophosphatemia had a higher ICU 28-day mortality than the normal control group (33.3% vs 24.0%, *P* < 0.05). Patients with hypophosphatemia had a longer ICU and hospital stays, and prolonged duration of mechanical ventilation (all *P* < 0.05). Hypophosphatemia was an independent risk factor for ICU 28-day mortality (adjusted OR = 1.5, 95% CI = 1.1–2.1, *P* = 0.01) in the multivariate logistic regression analysis.

**Conclusions:**

Hypophosphatemia at admission is an independent risk factor for 28-day mortality in general ICU patients.

**Trial registration:**

The medical study was approved by the Institutional Ethics Committee of the Six Affiliated Hospital, Sun Yat-sen University (Approval number: 2017ZSLYEC-110). No consent was given as the data were analyzed anonymously.

## Background

Phosphate is a vital component of the lipid bilayer in the cell membrane, which has essential functions in many biological processes, such as adenosine triphosphate production, glycolysis, pH buffering, 2,3-diphosphoglycerate (2,3-DPG) synthesis, mitochondrial functions, enzyme regulation, signal transduction, and nucleotide metabolism [1–3]. Furthermore, phosphorus is a source of ATP (adenosine triphosphate), required for the normal neurologic function and muscular contraction. Maintaining normal serum phosphate levels is extremely important as phosphate supply disturbance can result in multiple organ system dysfunction, which is not limited to systems, such as the respiratory [4–6], cardiac [7–9], immunologic [10], hematologic [11], or neuromuscular [12].

Phosphate homeostasis is complex, and the phosphate ion may be influenced by other factors, such as decreased renal clearance, increased consumption in catabolic patients, intestinal losses, or clearance over a continuous renal replacement therapy (CRRT) membrane. Importantly, hypophosphatemia is among the most frequently encountered electrolyte metabolic disturbances in critically ill patients with an incidence and prevalence ranging within 2.4–100% [13, 14]. Unlike other electrolyte disturbances, negligible importance has been given to the derangement of phosphate homeostasis during management of critically ill patients. Hypophosphatemia causes diverse clinical manifestations, including myocardial dysfunction, diaphragmatic weakness, seizures, coma, rhabdomyolysis, and red blood cell dysfunction due to tissue hypoxia (by decreasing erythrocyte 2,3-DPG) and impaired cellular energy stores. Therefore, the detection of phosphate metabolism abnormalities in Intensive Care Unit (ICU) populations is crucial.

Hypophosphatemia is often not diagnosed, because it remains asymptomatic, frequently presenting as fatigue and irritability. Reportedly, hypophosphatemia is associated with longer ICU and hospital stays [10, 15–17], increased risk of arrhythmia, and respiratory muscle dysfunction [18, 19]. Nonetheless, whether hypophosphatemia is associated with mortality in the general ICU patients is still debatable.

Although many studies have discovered that episodes of hypophosphatemia during the ICU stay is associated with increased mortality, whether it directly leads to higher mortality or is merely a marker of disease severity in the general ICU still remains uncertain. Few studies have been carried out to investigate the association between the serum phosphorus levels at admission and the outcomes of general ICU population. We speculated that hypophosphatemia would have an unfavorable impact on ICU 28-day mortality and can be a marker of disease severity. To confirm this assumption, a single-center retrospective cohort study was conducted to determine whether patients with hypophosphatemia had higher mortality than patients with normal serum phosphate levels. Our primary study endpoint was patient mortality on the 28th day.

## Methods

The study was conducted from 1 January 2016 to 31 December 2017 after approval by the Institutional Ethics Committee of the Six Affiliated Hospital, Sun Yat-sen University. A waiver for informed consent was obtained for this retrospective cohort study as data were analyzed anonymously. The data can be accessed with permission from the Institutional Ethics Committee of the Six Affiliated Hospital, Sun Yat-sen University by contacting the corresponding author. A total number of 946 patients were included at ICU admission. The following inclusion criteria were used: (1) age ≥ 18 years; (2) patients with serum phosphate level measured at admission at ICU; (3) survival status for a follow-up period of 28 days after ICU admission. The criteria for exclusion were: (1) age below 18 years; (2) pregnancy; (3) patients with a hospital stay of > 180 days; (4) patients who could not be followed up; (5) patients without serum phosphate level measured at admission to ICU; (6) patients with hyperphosphatemia (serum phosphate levels > 1.60 mmol/L).

A number of 1073 of patients were identified and screened, and 127 patients were excluded, resulting in a cohort of 946 patients finally included in our investigation.

### Collection of clinical and biochemical data

Variables were obtained from electronic medical records using the patient’s hospital admission number. The medical record of each patient who met the inclusion/exclusion criteria were reviewed to collect the following data: (1) age, (2) sex, (3) reason for ICU admission,(4) underlying disease,(5) medications, (6) nutrition, (7) Acute Physiology and Chronic Health Evaluation (APACHE II), levels of (8) phosphate, (9) potassium, (10) creatinine, (11) serum calcium, (12) serum albumin, and (13) glomerular filtration rate (GFR) patient admission to the ICU; the medical treatment included (14) mechanical ventilator time and (15) renal replacement therapy time. The parameters of the clinical outcomes included ICU’s length of stay, hospital’s length of stay, and ICU 28-day mortality. Parameters were obtained from electronic medical records by two independent, well-trained researchers. The data for the 28-day mortality in ICU was confirmed by telephone follow-up and inspection of the electronic medical data.

All phosphate level data were collected by the Laboratory for Clinical Chemistry at the Sixth Affiliated Hospital of Sun Yat-sen University using phosphomolybdate method by Beckman AU 5800. The reference range of phosphate was 0.80–1.60 mmol/L.

Phosphate concentrations at admission to the ICU were obtained, and the patients were divided into two groups. Patients with serum phosphate concentrations between 0.80 and 1.60 mmol/L were included in the normal control group, whereas those with phosphate concentrations lower than 0.8 mmol/L composed the hypophosphatemia group.

### Statistical analysis

Statistical analysis was conducted by SPSS 20.0 software. Continuous variables are expressed as mean and SD or median with interquartile range, depending on the underlying data distribution. Categorical variables are reported as percentages and frequencies. The continuous variables for age were compared by the APACHE II scores using the Student’s *t*-test. The other variables were analyzed using the Mann-Whitney U-test; categorical variables were compared through the chi-square test. Binary logistic regression analysis was employed to assess the association of variables with ICU 28-day mortality. Multivariate logistic regression was utilized to identify the risk factors for 28-day ICU mortality. *P* < 0.05 was considered statistically significant.

## Results

### Population characteristics

**Fig. 1:**
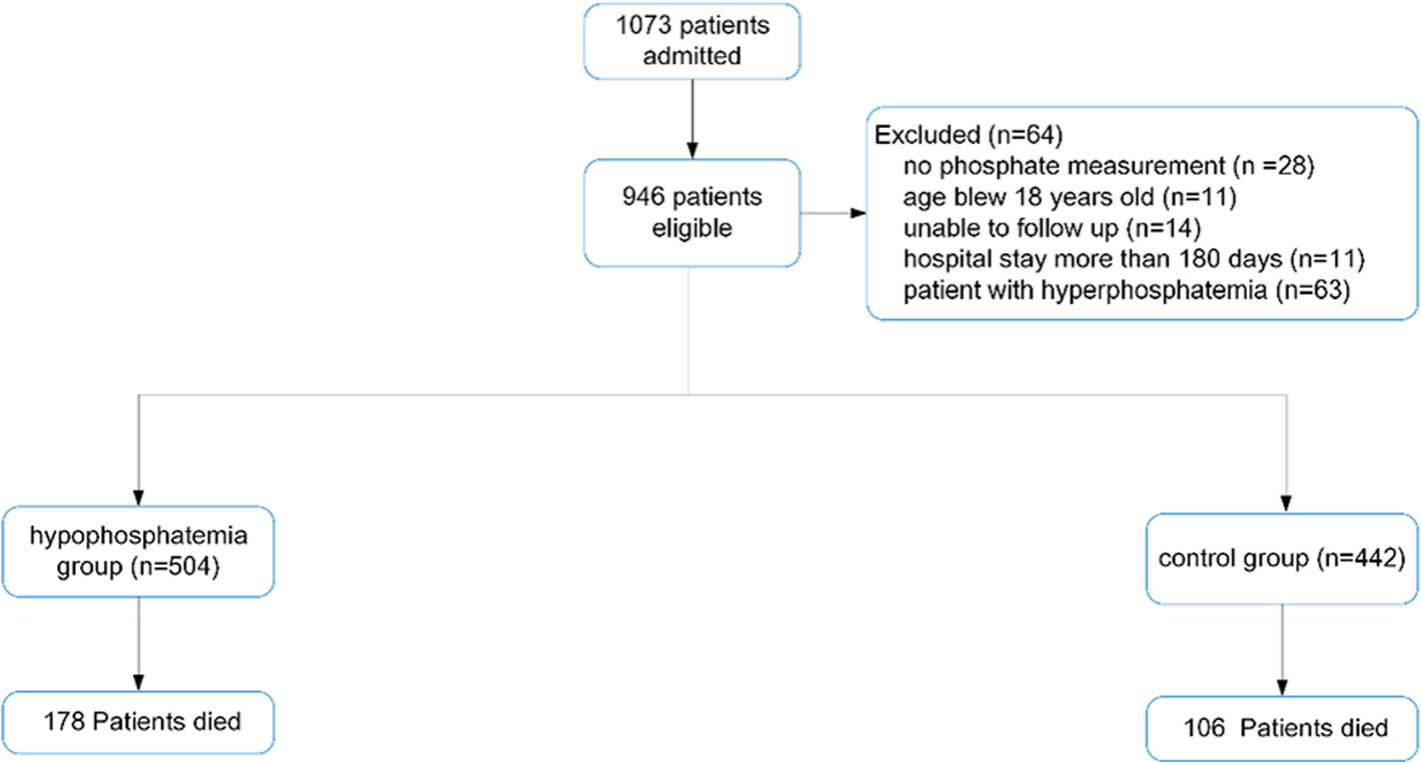
Patient flow chart

A total number of 1073 of adult patients were admitted to the general ICU from 2015 to 2016, of which 946 patients met the inclusion criteria. Of the total cohort, 504/946 (53.27%) and 442/946 (46.73%) were classified into the hypophosphatemia group and the normal phosphate group, respectively (Fig. 1).

The baseline characteristics of the patients are listed in Table 1. The patients were 62.41 ± 17.06 years old; 65.3% were male. The mean age of the patients and the sex distribution in the hypophosphatemia group was not significantly different from those in the normal phosphate group. These patients had high APACHE II scores (18.8 ± 7.6), and their 28-day in ICU mortality was 32.9%. The most common reasons for ICU admission were post-operation monitoring and sepsis, with an incidence of 35.2 and 29.7%, respectively. There were no significant differences between two groups in ICU admission reason and underlying disease. Medications and nutritional support were similar between the two groups. As can be seen in Table 1, the levels of serum albumin and serum potassium in the hypophosphatemia group were lower than those in the control group, but no difference was detected in the serum calcium, creatinine levels and GFR.

**Table 1:** Clinical characteristics of critically ill patients presented to the Six Affiliated Hospital, Sun Yat-sen University from 2016 to 2017

	Total	Control	Hypophosphatemia	*P*
Age (years), mean ± SD	62.41 ± 17.06	62.26 ± 17.32	62.58 ± 16.78	0.77
Male (%)	618 (65. 3%)	280 (63.6%)	338 (67.1%)	0.25
APACHE II scores, mean ± SD	18.78 ± 7.56	17.02 ± 7.43	25.64 ± 12.31	0.00
Reason for admission (%)				
Sepsis and sepsis shock	281 (29.7%)	125 (28.3%)	156 (31.0%)	0.37
Postoperative	333 (35.2%)	170 (38.5%)	163 (32.3%)	0.05
Hemorrhagic shock	64 (6.8%)	32 (7.3%)	32 (6.3%)	0.59
Respiratory failure	82 (8.7%)	38 (9.4%)	44 (8.7%)	0.94
Cardiovascular disease	66 (7.0%)	28 (6.3%)	38 (7.5%)	0.47
Cerebral disease	41 (4.3%)	21 (4.8%)	20 (4.0%)	0.56
Severe acute pancreatitis	8 (0.8%)	2 (0.5%)	6 (1.2%)	0.22
Others	71 (7.5%)	26 (5.9%)	45 (8.9%)	0.08
Underlying disease				
Cancer	587 (62.1%)	286 (64.7)	301 (59.7%)	0.12
Hypertension	181 (19.1%)	70 (15.8%)	111 (22.0%)	0.10
Diabetes mellitus	91 (10%)	40 (9.0%)	51 (10.1%)	0.58
Coronary heart disease	49 (5.2%)	23 (5.2%)	26 (5.2%)	0.98
Chronic renal insufficiency	74 (7.9%)	27 (6.1%)	47 (9.3%)	0.06
Chronic obstructive pulmonary disease	30 (3.2%)	12 (2.7%)	18 (3.5%)	0.45
Medications (%)				
Insulin	59 (6.3%)	25 (5.7%)	34 (6.8%)	0.49
Furosemide	22 (2.3%)	7 (1.6%)	15 (3.0%)	0.16
Glucocorticoid	24 (2.5%)	8 (1.8%)	16 (3.2%)	0.18
Nutrition (%)				
Parenteral	261 (27.6%)	120 (27.1%)	141 (28.0%)	0.78
Enteral	138 (14.6%)	70 (15.8%)	68 (13.5%)	0.39
Parenteral+ Enteral	240 (25.4%)	104 (23.5%)	136 (26.8%)	0.22
No nutrition support therapy	307 (32.5%)	148 (33.5%)	159 (31.5%)	0.52
Albumin (g/L), median (IQR)	31.2 (25.9, 36.8)	32.7 (27.3, 38.8)	30.0 (25.2, 35.0)	0.00
Calcium (mM), median (IQR)	1.99 (1.84, 2.14)	1.98 (1.84, 2.13)	2.00 (1.84, 2.15)	0.37
Potassium (mM), median (IQR)	3.9 (3.5, 4.3)	4.0 (3.6, 4.3)	3.8 (3.5, 4.3)	0.00
Creatinine (μM), median (IQR)	76.0 (54.0,124.0)	75.0 (55.0,113.0)	77.5 (52.5, 132.0)	0.63
Phosphorus (mM), median (IQR)	0.77 (0.55, 1.03)	1.05 (0.92,1.24)	0.56 (0.43, 0.67)	0.00
GFR (ml/min1.73m^2^)	76.36 ± 37.55	76.95 ± 36.35	75.84 ± 38.60	0.65

*SD* = standard deviation, *APACHE II* = Acute Physiology and Chronic Health Evaluation, *IQR* = interquartile ranges, *GFR* = glomerular filtration rate

### Clinical outcomes

As can be seen in Table 2, the hypophosphatemia group had higher 28-day ICU mortality (35.3% vs 24.0%, *P* < 0.05) than the normal control group. Additionally, the patients in the hypophosphatemia group had a longer ICU stay with a median of 5.5 (lower and upper quartiles; 2.0 and 10.6 days) than those in the control group, the median of which was 1.7 (lower and upper quartiles; 1.5 and 3.4 days), *P* < 0.05. Similar results were obtained for the length of hospital stay.

**Table 2:** Association of hypophosphatemia with clinical outcomes

	Total	Control	Hypophosphatemia	*P*
28-day in ICU mortality (%)	284 (30.0%)	106 (24.0%)	178 (35.3%)	0.00
ICU LOS (days), median (IQR)	3.4 (1.7, 6.7)	1.7 (1.5, 3.4)	5.5 (2.8, 10.6)	0.00
Hospital LOS (days), median (IQR)	23.5 (13.6, 37.6)	20.6 (12.6,31.4)	27.1 (15.6, 27.1)	0.00
Proportion of MV (%)	526 (55.6%)	172 (38.9%)	354 (70.23%)	0.00
MV (days), median (IQR)	3.0 (1.0, 6.8)	1.2 (0.7,2.9)	4.4 (2.9, 9.0)	0.00
Proportion of RRT (%)	133 (14.1%)	39 (8.8%)	94 (18.7%)	0.00
Duration of RRT (h), median (IQR)	66 (35,141)	41 (22.0,59.0)	81.0 (45.3188.8)	0.03

*ICU* = Intensive Care Unit, *LOS* = length of stay, *IQR* = interquartile ranges; *MV* = mechanical ventilation, *IQR* = interquartile ranges, *CRRT* = continuous renal replacement therapy

**Fig. 2:**
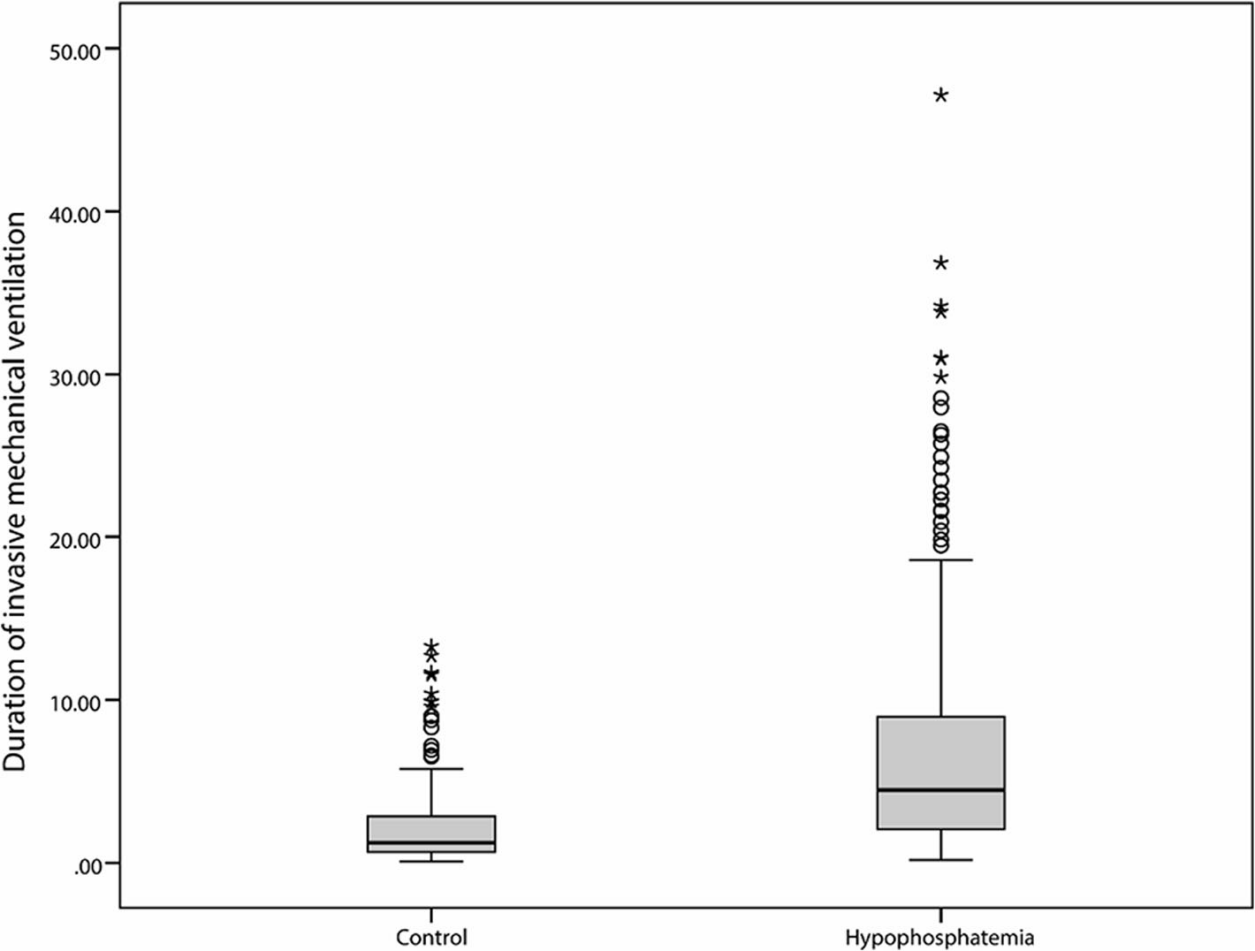
Duration of invasive mechanical ventilation

A total number of 526 of 946 (55.6%) patients received ventilation. Significantly higher mechanical ventilation was applied for the patients in the hypophosphatemia group as compared to those in the normal control group (70.23% vs 38.91%, *P* < 0.05). As illustrated in Fig. 2, the hypophosphatemia group manifested remarkably longer ventilator time with a median of 4.4 (lower and upper quartiles; 2.9 and 9.0 days) than the normal control group, the median of which was 1.2 days (0.7 and 2.9 days).

As showed in Table 2, one hundred and thirty-three of 946 (14.1%) patients got RRT. The hypophosphatemia group had significantly longer time on CRRT when compared to control group, a median of 81.0 (lower and upper quartiles; 45.3and 188.8 h) compared to 41 h (22.0 and 59.0 h) in the normal control group.

### Predictors of mortality

The association of different laboratory parameters with ICU 28-day mortality is presented in Table 3. Binary logistic regression analysis results revealed that the APACHE II score, male gender, serum albumin level, and hypophosphatemia were associated with ICU 28-day mortality, but age, serum potassium, serum calcium, and creatinine were not. After adjustment for APACHE II scores, serum albumin and gender, multivariable logistic regression analysis showed that hypophosphatemia (Table 3) was significantly associated with an increased risk of ICU 28-day mortality (OR = 1.5, 95% CI =1.1–2.1, *P* = 0.01).

**Table 3:** Unadjusted and adjusted analysis of factors associated with 28-day mortality in ICU

	Unadjusted OR (95% CI)	*P*	Adjusted OR (95% CI)	*P*
Age	(1.0,1.0)	0.13	–	–
Male (%)	1.5 (1.1,2.2)	0.02	0.7 (0.5,0.9)	0.02
APACHE II	1.2 (1.1,1.2)	0.00	1.2 (1.1,1.2)	0.00
Calcium	1.7 (0.9,3.4)	1.12	–	
Potassium	1.2 (0.9,1.5)	0.17	–	–
Creatinine	1.0 (1.0,1.0)	0.85	–	–
Albumin	0.9 (0.9,1.0)	0.00	0.9 (0.9,1.0)	−0.00
Hypophosphatemia	1.6 (1.1,2.2)	0.01	1.5 (1.1,2.1)	0.01

## Discussion

Although previous studies have indicated that hypophosphatemia is associated with worse clinical outcomes in critically ill patients [14, 20], few studies have been carried out to investigate the association between the serum phosphorus levels at admission and the outcomes of general ICU population. Whether hypophosphatemia itself causes higher mortality or is a marker of illness severity remains unclear.

Earlier evidence has revealed that episodes of hypophosphatemia during the ICU stay is associated with higher mortality in the ICU population [10, 15–17]. After the adjustments for confounding factors, Marcus Broman [15] found that when compared with normal phosphate group, patients with hypophosphatemia episodes had higher risk of death (HR = 1.2, 98.3% CI = 1.0–1.5, *P* = .0089). However, they defined hypophosphatemia as phosphate levels < 0.7 mmol/L and no phosphate levels > mmol/L during the ICU stay. A retrospective study was conducted by Yi Yang [17] on patients who had developed hypophosphatemia during the CVVH therapy period. They divided the patients into two groups, the ratio of hypophosphatemia to total CVVH therapy days lower than 0.58 defined as low ratio group, and the other as high ratio group. They found that, compared to the low ratio group, the high ratio group had a 1.451-fold in 28-day mortality rate (95% CI 1.103–1.910, *P* = 0.008). Renana Shor [16] reported that sever hypophosphatemia indicated a higher mortality than those without severe hypophosphatemia (80.8% VS 34.5%, *P* = 0.001). But unlike our study, they selected septic patients for study subjects, with a small population size. On the other hand, Dominik [21] and Satoshi [20] found that hypophosphatemia was associated with a longer hospital stay, but was not an independent predictor of mortality in the ICU population. The association between serum phosphate abnormalities and the clinical prognosis is still debatable. We conducted the study in the general ICU, most of our patients admitted to ICU for post-operation monitoring and sepsis. We found that hypophosphatemia at ICU admission was related to longer ICU and hospital stays, and hypophosphatemia was associated with higher mortality. The results of the multivariable logistic regression analysis showed that hypophosphatemia (< 0.80 mmol/L) can predict 28-day mortality in ICU.

We found that male sex, APACHE II scores, serum albumin level and hypophosphatemia were associated with 28-day mortality in the general ICU population. Then, we examined whether the association between phosphorus levels and 28-day mortality were related to the illness severity and the nutritional status. Even after adjustment for APACHE II scores and serum albumin, and the gender, hypophosphatemia (< 0.80 mmol/L) was still associated with increased 28-day mortality. This indicates that hypophosphatemia (< 0.80 mmol/L) was associated with the 28-day mortality regardless of the illness severity and nutritional status in the general ICU.

In the present investigation, we established that hypophosphatemia was associated with an increased duration of mechanical ventilation. As Christopher [22] reported, the duration of ventilation in the hypophosphatemia group was 3.0 [1.7–5.9] days, whereas in the normophosphatemia group it was 4.8 [2.3–10.5] days. Phosphorus is a source of ATP (adenosine triphosphate) required for neurologic functions and muscular contraction. Phosphate supply disturbance can lead to multiple organ system dysfunctions, included respiratory failure [3, 4]. As established in our study, the patients in the hypophosphatemia group required more intensive and prolonged mechanical ventilation.

Our study showed that the hypophosphatemia group had a higher proportion of RRT than that in the normophosphatemia group (8.8% VS 18.7%, *P* = 0.00), and the former had a longer duration of RRT time. Renal replacement therapy (RRT) has been recommended for severe renal failure in critically-ill patients, such as those patients with sepsis shock and complicated with acute renal failure (ARF). RRT is also used in patient with chronic renal failure in the situation of hemodynamic instability. In our study population, although there was no difference between the two groups in the reasons for ICU admission, but the number of septic shock patients complicated with acute renal insufficiency and received RRT therapy in hypophosphatemia group is higher than that in the control group (22.4% VS 36.5%, *P* = 0.01).

Hypophosphatemia may lead to a multitude of complications in critically ill patients [2, 10] since numerous cellular mechanisms require phosphates. Hypophosphatemia is associated with cardiac, respiratory, immunologic, and hematologic disorders, which is a subsequence of the impaired energy metabolism. Hypophosphatemia can be asymptomatic but may as well be accompanied by fatal clinical complications, leading to poor outcomes in critically ill patients.

Some important limitations to our study should be acknowledged. First, this investigation was retrospective and performed at a single medical center. Second, we identified only the baseline measurements of the plasma phosphate level, which hindered the evaluation of plasma phosphate values over time. Phosphate replacement [22] is recommended in symptomatic hypophosphatemia and phosphate levels < 0.32 mmol/L. Whether maintenance of normal plasma phosphate level and correction of the hypophosphatemia in critically -ill-patients can improve outcome is currently unknown. Further randomized controlled trials are required to assess the benefit when the patients are treated with hypophosphatemia. Finally, we did not have information on the other confounders, such as fluid therapy, but we had a large number of other confounding variables for adjustment. Therefore, further studies in larger populations are required to confirm our findings.

## Conclusions

Phosphate abnormality is common in ICU, and hypophosphatemia is an independent indicator of 28-day mortality in the general ICU population. The development of hypophosphatemia should be diagnosed early to prevent its harmful effects.

## Abbreviations

2, 3-DPG: 2, 3-diphosphoglycerate
APACHE II: Acute Physiology and Chronic Health Evaluation
ARF: Acute Renal Failure
CRRT: continuous renal replacement therapy
ICUs: Intensive Care Units
